# Fibroblast growth factor 8b induces uncoupling protein 1 expression in epididymal white preadipocytes

**DOI:** 10.1038/s41598-019-44878-w

**Published:** 2019-06-11

**Authors:** Sören Westphal, Thomas Gantert, Caroline Kless, Kristina Hüttinger, Martin Klingenspor, Tobias Fromme

**Affiliations:** 1 Department of Internal Medicine II, University of Ulm, Ulm, Germany; 2 Chair of Molecular Nutritional Medicine, TUM School of Life Sciences, Technical University of Munich, Freising, Germany; 3 EKFZ - Else Kröner-Fresenius Center for Nutritional Medicine, Technical University of Munich, Freising, Germany; 4 ZIEL - Institute for Food & Health, Technical University of Munich, Freising, Germany

**Keywords:** Cell biology, Fat metabolism

## Abstract

The number of brown adipocytes residing within murine white fat depots (brite adipocytes) varies a lot by depot, strain and physiological condition. Several endocrine fibroblast growth factors are implicated in the regulation of brite adipocyte abundance. The family of fibroblast growth factors can be categorized by their site of action into endocrine, paracrine and intracellular peptides. We here screened paracrine fibroblast growth factors for their potential to drive brite adipogenesis in differentiating epididymal white adipocytes and identified fibroblast growth factor 8b to induce uncoupling protein 1 expression, but at the same time to interfere in adipogenesis. In an *in vivo* trial, fibroblast growth factor 8b released into the epididymal fat depot failed to robustly increase the number of brite adipocytes. The specific action of fibroblast growth factor 8b on the uncoupling protein 1 promoter in cultured epididymal adipocytes provides a model system to dissect specific gene regulatory networks.

## Introduction

Brown adipose tissue (BAT) is an organ equipping mammals with a means of non-shivering thermogenesis. In brown adipocyte mitochondria, uncoupling protein 1 (Ucp1) allows re-entry of protons from the intermembrane space into the matrix bypassing ATP synthase and thus uncoupling oxygen consumption from ATP production. By this mechanism, the energy stored in the form of proton motive force is released as heat (for a review, see^1^).

BAT and its ability to combust nutrient energy into heat has gained raising attention by the scientific community after the repeated and convincing demonstration that healthy human adults possess appreciable amounts of metabolically active BAT^2–4^. Physiological or pharmacological activation of BAT thermogenesis may prove effective in treating some of the most widespread diseases of our time including obesity, diabetes and dyslipidemia. The amount of human BAT, however, is limited and estimated to be in the range of 0.5% of the body mass as compared to a more than 10-fold higher amount in mice^5^. Thus, not only acute activators may be required to therapeutically employ the unique capabilities of BAT, but also agents that recruit a greater number of brown adipocytes.

Brown adipocytes are not restricted to uniform classical BAT depots but are often found interspersed in otherwise white adipose tissue (WAT) depots. This second type of brown adipocyte has been termed beige or brite (brown in white) and seems to emerge from a different progenitor cell than classical brown fat cells (reviewed in^6^). To convert WAT into BAT by means of recruiting brite cells offers a possibility to massively increase the BAT amount accessible to therapeutic activation and at the same time would decrease the amount of WAT, thereby replacing an energy-storing organ with an energy-dissipating one. This browning of white fat has been subject of intense research and several systemic interventions are known to increase at least to a certain degree the number of brite cells in mice, including cold exposure and treatment with beta-adrenergic agonists, cardiac natriuretic peptides or fibroblast growth factor 21 (FGF21)^7–11^.

The effectiveness of FGF21 prompted us to investigate further members of the fibroblast growth factor (FGF) family. FGFs can be grouped by their mechanism of action into intracellular, paracrine and endocrine peptides. FGF21 belongs to the small group of endocrine FGFs and is therefore able to exhibit systemic effect on multiple target tissues. The largest group is formed by the paracrine FGFs which feature a protein domain binding to extracellular matrix components and are thereby less mobile and not found in circulation^12^. Their matrix anchor also serves to stabilize interaction with FGF receptors, while endocrine FGFs require an additional cofactor of the klotho family for that purpose. Paracrine FGFs can be expected to act locally on the target tissue they are released into. We investigated the potential of paracrine FGFs to re-route the differentiation of white preadipocytes towards brite mature adipocytes in the present study.

## Material & Methods

### Fibroblast growth factors

We obtained fibroblast growth factors (FGFs) 1, 2 and 9 of murine origin and human FGFs 5 and 16–21 from PeproTech. The murine FGFs 4, 6, 7, 8b, 10, and 23 and human FGFs 3, 8a, 8e, 8f and 22 were purchased from R&D Systems. The concentration used for screening purposes in cell culture was as follows (ng/ml): FGF1 2.5, FGF2 5.0, FGF3 25.0, FGF4 5.0, FGF5 2.5, FGF6 5.0, FGF7 25.0, FGF8b 25.0, FGF9 1.0, FGF10 50.0, FGF16 2.5, FGF17 10.0, FGF18 5.0, FGF20 10.0 and FGF22 125.0. The concentration chosen for each factor was based on the specific biological EC_50_ value determined in fibroblast proliferation assays by the respective supplier.

### Cell culture

Preadipocytes were isolated from the stromal vascular fraction of inguinal or epididymal white adipose tissue of newborn wild-type mice of the FVB strain. Cells were immortalized by infection with a puromycin resistance-conferring retroviral vector encoding the Simian Vacuolating Virus 40 large T antigen (SV40 T-antigen) and selected with puromycin as published previously^13,14^. Cells were grown to confluence in Dulbecco’s modified Eagle medium (4.5 g/l glucose, GE Healthcare Bio-Sciences) supplemented with 20% fetal bovine serum (Life Technologies), 20 nM insulin and 1 nM T3. Adipocyte differentiation was induced by complementing this medium with 250 µM indomethacin, 500 µM isobutylmethylxanthine and 2 µg/ml dexamethasone for 24 h (48 h for respirometric plates) after confluence. Cell culture was continued for up to six more days.

Glucose uptake was determined with a commercial system based on the detection of 2-deoxyglucose-6-phosphate following the manufacturer’s protocol (Glucose Uptake-Glo, Promega). Lactate concentration was measured with an established assay based on the spectrophotometric detection of NADH at 340 nm formed by lactate dehydrogenase (Roche) in the presence of hydrazine at alkaline pH^15^. Glycerol in cell culture supernatant was measured with Free Glycerol Assay Kit (ab65337, Abcam). Size distribution of lipid droplets was evaluated by unstained, automated image analysis (WimLipid algorithm, Wimasis/Onimagin). Proliferation of preadipocytes was assessed by using a resazurin-based assay (CellTiter-Blue, Promega). Cultured cells were incubated with the reagent for 4 hours with standard culture conditions. Fluorescence of collected media was measured at 560Ex/590Em. Lipid droplets of differentiated adipocytes were stained with 3 µg/ml of the fluorescent lipophilic dye 4,4-difluoro-3a,4a-diaza-s-indacene (BODIPY 493/503) in PBS for 15 min, then washed with PBS and imaged on a Leica DMI6000B fluorescence microscope under 20x magnification (EX/EM: 488–503/515–545).

### Respirometry

Oxygen consumption and extracellular acidification was measured in collagen-coated 96-well format plates on a Seahorse XF96 Extracellular Flux Analyzer (Agilent Technologies). Culture medium was substituted for assay medium (DMEM Base D5030, 5 mM Hepes, 25 mM glucose, 31 mM NaCl, 2 mM GlutaMAX™ (Gibco), 0.1% (w/v) free fatty acid free bovine serum albumin (BSA), 15 mg/L phenol red and adjusted to pH 7.4 at 37 °C) 1 h prior to measurement. Ucp1-mediated respiration was measured as published previously.^16,17^ Briefly, after basal conditions we added 5 µM oligomycin, 1 µM isoproterenol, 7.5 µM FCCP and 5 µM antimycin A. Maximum glycolytic capacity was determined as published previously^18^. Briefly, basal glycolytic rate was measured after the addition of 10 mM glucose enabling ATP production by both oxidative phosphorylation and glycolysis. Subsequently, respiration was inhibited by 1 µM rotenone and 5 µM antimycin A increasing glycolytic flux. To assess maximum glycolytic capacity, cellular ATP demand is increased by the addition of 400 µM monensin and 2 µM FCCP.

### Quantitative PCR

Total RNA was isolated using the Qiazol reagent (Qiagen). Quality of RNA was tested by photometric analysis and agarose gel electrophoresis. 5 µg of total RNA were reverse transcribed using the iScript cDNA Synthesis Kit (Biorad) in a 20 µl reaction. Target mRNAs were amplified in a total volume of 25 µl containing iQ SYBR Green Supermix (Biorad) and 10 pmol of each primer using the Mastercycler realplex 2 detection system (Eppendorf). Transcript abundance was normalized to the expression of stable housekeeping gene transcripts as provided in the figure legends. Primer sequences are provided in Supplemental Table 1.

### Western blot

Sample protein was isolated using RIPA buffer (50 mM Tris-Cl, 1% (v/v) NP-40, 0.25% (w/v) Na-deoxycholate, 150 mM NaCl, 1 mM EDTA, 1:1000 protease inhibitor (P8340-1 ml, Sigma)). Protein concentrations were determined with Pierce™ BCA Assay Kit (Thermo Scientific). Equal amounts of protein in Laemmli loading buffer (33 mM Tris-HCI, pH 6.8, 5% SDS, 25% (w/v) glycerol, 0.01% bromophenol blue) were run on 4–20% Mini-PROTEAN® TGX Stain-Free™ Precast Gels (Biorad), blotted onto nitrocellulose membranes, blocked in 3% (w/v) bovine serum albumin in Tris-buffered saline (TBS) and incubated overnight with primary antibodies rabbit Anti-UCP1 (ab23841, Abcam), rabbit Anti-Perilipin-1 (D1D8) XP® (#9349, CST), rabbit Anti-HSL (#4107, CST), mouse Anti-β-Actin or clone C4 (MAB1501, Merck Millipore). Membranes were washed three times with TBS-0.1% Tween20 before and after incubation with secondary antibodies IRDye® 800CW goat anti-rabbit and IRDye® 680CW donkey anti-mouse for 1 h at RT. Signals were detected by the Odyssey Infrared Imaging System (LI-COR). Image analysis was performed using the Image Studio™ Lite Software (LI-COR).

### Pharmacological treatment

All animal experimentation was conducted according to the German Animal Welfare Act and previously approved by the relevant authority (Government of Upper Bavaria, file no. 55.2-1-54-2532-174-11). We employed male mice of the 129S6/SvEvTac inbred strain bred in our specified pathogen-free facility at 50–60% relative humidity, 22 °C ± 1 °C and a 12-hour light/dark cycle. All mice received standard rodent chow diet (V1124-300, Ssniff Spezialdiäten GmbH). Implantable, bio-degradable pellets (1.5 mm diameter) were custom designed to continuously release 100 ng paracrine Fgf8b per day into the surrounding interstitium for 3 weeks (Innovative Research of America). We implanted one pellet each unilaterally into the epididymal white adipose tissue depots of male 129SvEv mice in three independent experimental cohorts. After three weeks, all mice were sacrificed, adipose tissues samples and analyzed. In cohort 1, we also determined basal metabolic rate by indirect calorimetry. To measure maximal thermogenic capacity, we injected 1 µg norepinephrine per g body mass and monitored heat production for another 2 hours. In cohort 2, harvested adipose tissue depots were used for histology and UCP1 immunostaining. In cohort 3, sampled adipose tissues were analyzed by enzyme activity assays. We took photographs of the epididymal depot of all animals. Cropped images were evaluated by six independent, blinded observers attributing a number between 1 (not brown at all) and 10 (very brown) to the pellet implantation site. Plasma metabolites were quantified with a combined analytical test (Piccolo Lipid Panel Plus, Abaxis, USA).

### Immunostaining and enzyme activity assays

Dissected adipose tissues were fixed in phosphate buffered saline (PBS) with 4% paraformaldehyde/0.0024% picric acid and subsequently dehydrated, paraffin-embedded and cut into 5 µm sections. For immunohistochemistry, sections were cleared from paraffin, and subjected to sodium citrate-mediated epitope retrieval at 90 °C for 30 minutes and further incubated in 3% H_2_O_2_ for 10 minutes. Sections were blocked in PBS/2.5% normal goat serum prior to the overnight-incubation of the primary antibody at 4 °C (1:500 rabbit anti UCP1 (Abcam, ab10983) in PBS plus 0.1% Tween-20 and 0.25% normal goat serum. After secondary antibody (1:200, goat anti-rabbit, Abcam, ab97051) incubation for 1 hour, we rinsed thrice with PBS/0.1% Tween-20, and incubated 2 minutes with diaminobenzidine solution (DAB Enhanced Liquid Substrate System, Sigma-Aldrich). Images of mounted sections were taken with a bright field microscope using similar adjustments for all slides. Thirty sections per depot were evaluated by six independent, blinded observers who categorized each section by number of multilocular adipocytes (none, few, some, many) and their immunostaining (yes, no).

For respirometry, we digested minced adipose tissue in HBSS with 1 g/L collagenase. Swimming, lipid-laden adipocytes were transferred into MiR05 buffer^19^ in a Clark-type electrode (Rank Brothers), permeabilized by 4 µM digitonin and measured in the presence of 5 µM rotenone and 10 mM glycerol-3-phosphate. Cytochrome C oxidase activity was determined as described previously in the presence of 10 µM cytochrome C^20^. Citrate synthase activity was determined as described previously^21^.

### Statistical analyses

Data are presented as mean values ± standard deviation. All analyses have been performed with Prism 6 (Graphpad). Group differences were assessed by t tests with a significance level of 0.05. In the case of multiple group comparisons within a dataset we employed the appropriate adjustments (Tukey’s or Holm-Sidak’s multiple comparisons test). To compare metabolic states during extracellular flux assays, we selected the last basal measurement (basal respiration, basal extracellular acidification rate) and the first measurement after injection for all other states.

## Results

### Identification of FGF8b in a screen of paracrine fibroblast growth factors

Paracrine fibroblast growth factors are encoded by a gene family of 15 members designated Fgf1–10, Fgf16–18, Fgf20 and Fgf22^12^. We obtained the respective peptides of recombinant murine or human origin to screen their potential to induce the expression of the brown adipocyte specific gene uncoupling protein 1 (Ucp1) in white adipocytes. We chose immortalized white adipocytes established from primary stromal-vascular cells isolated from the murine epididymal adipose tissue depot^13,14^. The visceral, epididymal adipose tissue depot is considered to be homogenously white and to contain only a small number of brite adipocytes in mice^22–24^.

The treatment of cells was started after induction and continued during the differentiation period of 6 days. The concentration chosen for each factor was based on the specific biological EC_50_ value determined in fibroblast proliferation assays by the supplier and ranged between 1 and 50 ng/ml, as specified above.

**Figure 1:**
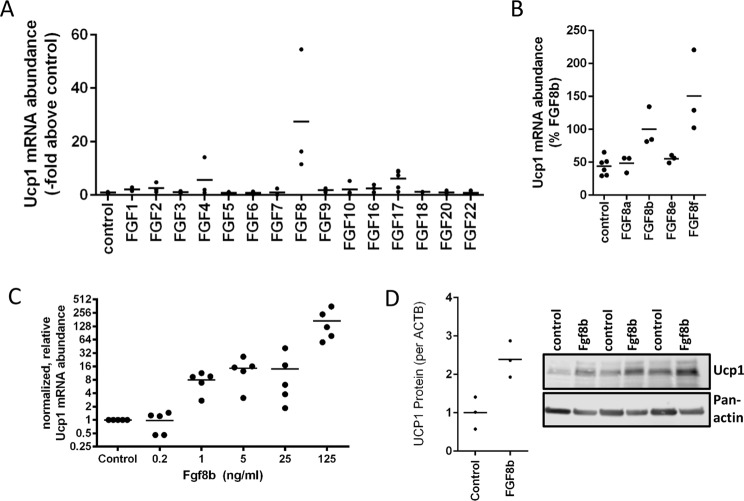
Paracrine fibroblast growth factor 8b enhances Ucp1 mRNA und protein expression in white adipocytes. (**A**) Screening of all paracrine FGFs in immortalized epididymal white adipocytes. FGF8b and FGF17 led to increased expression of Ucp1 mRNA expression, n = 3. (**B**) Peptide products of four different splice isoforms of the Fgf8 transcript were compared. Fgf8b and Fgf8f were able to increase Ucp1 transcript abundance, Fgf8a and e were not, n = 3. (**C**) Fgf8b dose dependently increased Ucp1 transcript abundance, n = 5. (**D**) The Fgf8b induced increase in transcript abundance was accompanied by increased Ucp1 protein. Depicted is a representative Western Blot and quantified signal intensities, n = 3.

Out of 15 tested fibroblast growth factors, Fgf8 induced strongest Ucp1 mRNA abundance (27.4-fold) (Fig. 1A). The numerical fold increase of Ucp1 expression in response to Fgf8 displayed a high variability in this and the following experiments mostly owing to very low Ucp1 transcript abundance in the control cells. The murine FGF8 gene gives rise to 8 differently spliced transcripts leading to 8 different peptide factors Fgf8a-f of which we initially tested the predominantly expressed splice form Fgf8b^25^. We compared the ability of those 4 murine isoforms that are also found in the human body (Fgf8a, b, e and f) to increase Ucp1 mRNA abundance (Fig. 1B). In epididymal adipocytes, Fgf8b and Fgf8f were both effective. For further experiments we chose the most abundant splice form Fgf8b with a completely identical amino acid sequence in mice and men.

**Figure 2:**
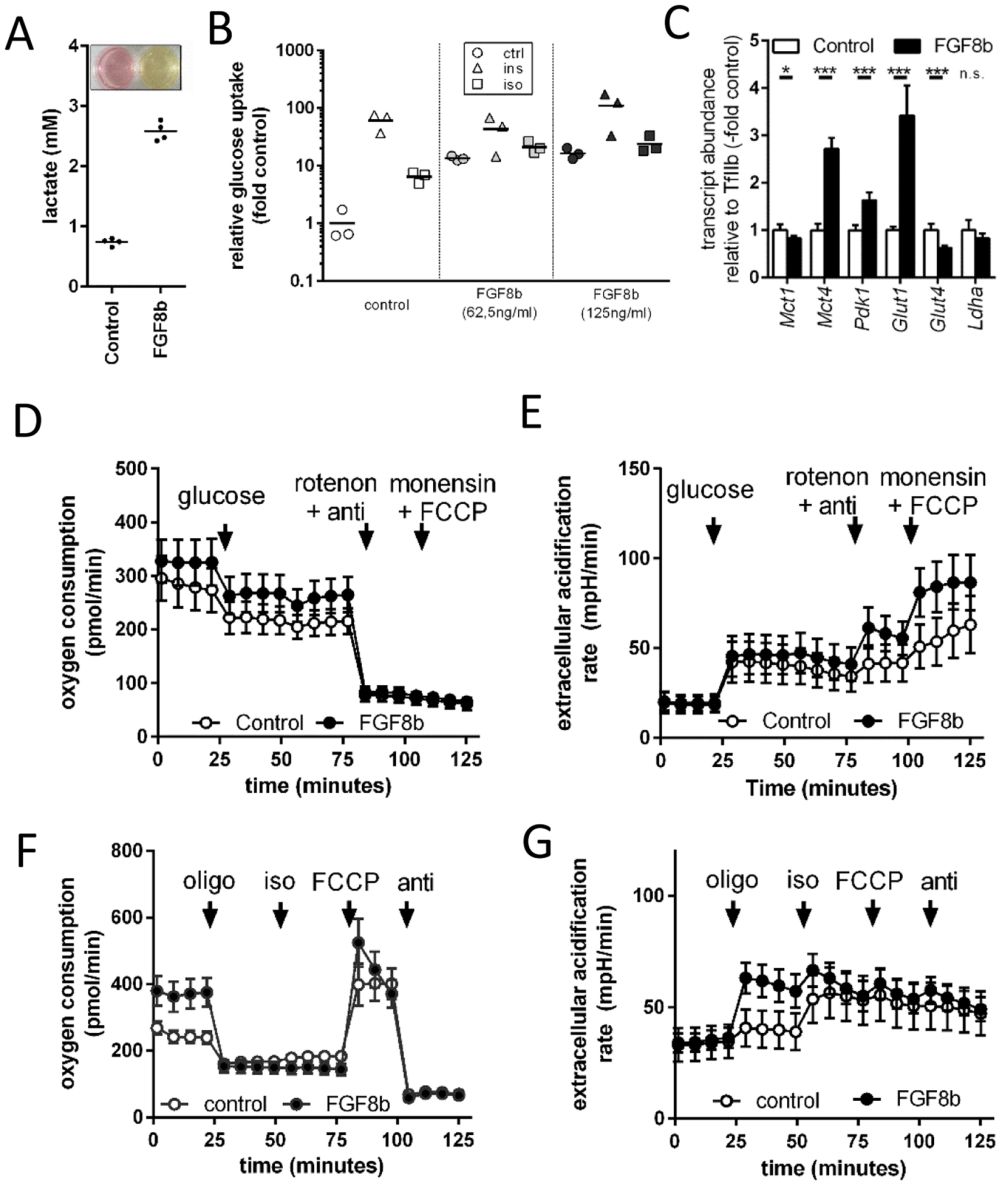
Metabolic alterations in Fgf8b treated adipocytes. (**A**) Adipocytes were treated with 125 ng/ml FGF8b or not (control). Depicted are lactate concentration and medium colour of treated versus untreated cells at the end of a six day differentiation course and 48 hours after a medium change. (**B**) Relative glucose uptake was measured in untreated (control) or Fgf8b treated (62.5 ng/ml and 125 ng/ml, respectively) adipocytes in response to insulin (ins, 20 nM, 10 min) or isoproterenol (iso, 500 nm, 10 min), n = 3. (**C**) Transcript abundance of genes associated with glycolysis. Lactate exporters monocarboxylate transporter 1 and 4 (Mct1, Mct4) as well as pyruvate dehydrogenase kinase 1 (Pdk1) and glucose transporter 1 (Glut1) were increased by Fgf8b treatment, while glucose transporter 4 (Glut4) was downregulated. Lactate dehydrogenase (Ldha) remained unchanged, n = 5, *p < 0.05, ***p < 0.001, Holm-Sidak multiple test. (**D**) Oxygen consumption of differentiated adipocytes treated for 24 hours with 125 ng/ml Fgf8b and measured in initially glucose free medium. Injections are indicated by arrows in this order: 10 mM glucose, 1 µM rotenone plus 5 µM antimycin A (anti), 400 µM monensin plus 2 µM FCCP. Shown are mean values ± SD n = 12–18. E Extracellular acidification rate of the experiment shown in (**D**). (**F**) Oxygen consumption of differentiated adipocytes treated for 5 days with 125 ng/ml Fgf8b and measured regular assay medium (25 mM glucose). Injections are indicated by arrows in this order: 5 µM oligomycin (oligo), 1 µM isoproterenol (iso), 7.5 µM FCCP, 5 µM antimycin A (anti). Shown are mean values ± SD, n = 30. (**G**) Extracellular acidification rate of the experiment shown in (**F**).

Murine, epididymal white adipose tissue is considered to be a classical white fat depot with little inclination to recruit interspersed brite adipocytes^26^. Treatment with Fgf8b, however, was able to increase Ucp1 mRNA abundance in a dose-dependent manner. This increase in Ucp1 transcript translated into an increase in Ucp1 protein (Fig. 1C,D). As was evident from the medium color after 48 h on fully differentiated adipocytes, Fgf8b treatment led to intense acidification of the medium indicative of a high metabolic rate and high glucose utilization (Fig. 2A). Indeed, glucose uptake was 10-fold higher after the treatment with Fgf8b and could be stimulated to a greater extent than controls by the β-adrenergic agonist isoproterenol (Fig. 2B). The transcript abundance of glycolysis associated genes was increased accordingly (Fig. 2C). A 24 hour treatment of differentiated adipocytes with 125 ng/ml Fgf8b was sufficient to increase metabolic rate as measured by basal oxygen consumption in glucose free medium (p < 0.05; Fig. 2D). Both control and treated adipocytes readily switched to more glycolytic ATP production upon addition of 10 mM glucose (Fig. 2D,E), but Fgf8b treated adipocytes displayed a far larger capacity to glycolytically compensate for a loss in mitochondrial ATP production and a higher maximal glycolytic flux rate (both p < 0.001; Fig. 2E)^18^.

Increased metabolic rate and increased Ucp1 abundance after several days of Fgf8b treatment seemed indicative of functional brite adipocytes. We measured Ucp1-mediated maximal respiration by an established assay^16,17^, but did not detect isoproterenol induced respiration – i.e. Ucp1 activity - in treated or control adipocytes (Fig. 2F). However, Fgf8b treated adipocytes displayed a much higher basal, but similar leak respiration compared to untreated controls, indicating a higher basal rate of ATP turnover (p < 0.001, Fig. 2F). This high basal ATP demand was provided by a higher glycolytic ATP production rate after inhibition of mitochondrial ATP synthesis by oligomycin (p < 0.001; Fig. 2G). Taken together, Fgf8b treatment increased metabolic rate and glycolytic flux rate and capacity, but did not lead to detectable activation of the increased amounts of Ucp1 by an adrenergic stimulus.

### Fgf8b interferes in adipogenic differentiation

**Figure 3:**
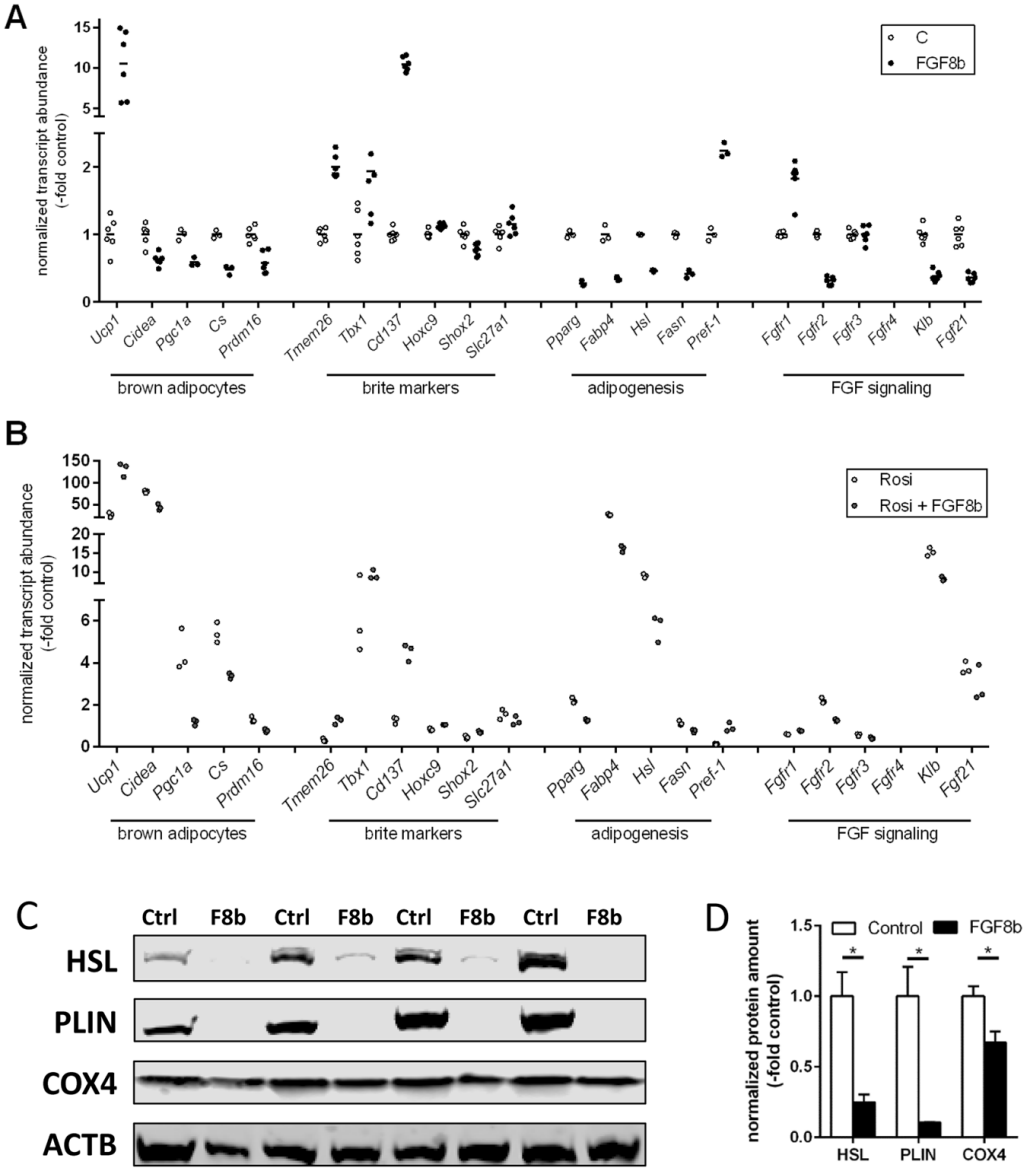
Gene expression profile of Fgf8b and PPARy agonist treated white adipocytes. White adipocytes were treated with Fgf8b (125 ng/ml) and/or rosiglitazone (20 µM) during differentiation. (**A**,**B**) Transcript abundance of genes indicative for brown adipocytes, brite markers, adipogenesis, and FGF signalling (abbreviations below), n = 3–6. Fgf8b treatment leads to significantly different abundance of all transcripts analysed except Tbx1, Slc21a1 and Fgfrs 3 and 4 both in the absence and in the presence of rosiglitazone and except Fgf21 in the presence of rosiglitazone (Holm-Sidak multiple testing). (**C**) Representative Western Blot detecting terminal adipogenic differentiation markers HSL and perilipin and mitochondrial marker COX4. (**D**) Quantified, normalized band intensity of proteins detected in (**C**), n = 3, bars represent mean values ± SD. Stars indicate a significantly different abundance of this transcript as compared to the control. Ucp1, uncoupling protein 1; Cidea, cell death-inducing DNA fragmentation factor alpha-like effector A; Pgc1a, peroxisome proliferator-activated receptor gamma, coactivator 1 alpha; Cs, citrate synthase; Prdm16, PR domain containing 16; Tmem26, Transmembrane protein 26; Tbx1, T-box transcription factor 1; Cd137, cluster of differentiation 137; Hoxc9, homeobox C9; Shox2, Short stature homeobox 2; Slc27a1, Long-chain fatty acid transport protein 1; Pparg, peroxisome proliferator-activated receptor gamma; Fabp4, fatty acid binding protein 4; Hsl, hormone-sensitive lipase; Fasn, fatty Acid Synthase; Pref-1, preadipocyte secreted factor-1; Fgfr, fibroblast growth factor receptor; Klb, klotho beta; Fgf21, fibroblast growth factor 21; HSL, hormone sensitive lipase; PLIN, perilipin; COX4, cytochrome C oxidase subunit 4.

We compared the browning potency of Fgf8b to a known, strong browning agent, the PPARγ agonist rosiglitazone. Fgf8b proved similarly effective and to act in a synergistic manner with rosiglitazone as far as UCP1 expression is concerned (Fig. 3A,B). Surprisingly, induction of the well characterized brown and brite adipocyte marker cell death-inducing DNA fragmentation factor alpha like effector A (Cidea), the master regulator of mitochondrial biogenesis PPARγ coactivator 1a (Pgc1a) and the mitochondrial marker citrate synthase (Cs) by rosiglitazone were all suppressed by the Fgf8b treatment (Fig. 3B). These observations seemed incompatible with the synergistic recruitment of brite adipocytes. Several transcripts described as specific brite markers (Tmem26, Tbx1, Cd137) or at least to be associated with brite cell containing fat depots^27,28^ were, however, increased by Fgf8b similarly to Ucp1.

Components of FGF signaling were differentially regulated by Fgf8b treatment with increased Fgfr1 transcript abundance, but decreased Fgfr2, the cofactor of endocrine Fgf binding beta-klotho and the endocrine Fgf21 (Fig. 3A). This pattern is markedly different in response to treatment with rosiglitazone (Fig. 3B). While Fgf21 and rosiglitazone synergistically interact in metabolic signaling^29,30^, Fgf8b rather counteracted rosiglitazone effects, indicating different signaling pathways downstream of Fgf21 and Fgf8b action in adipocytes.

In addition to brown adipocyte markers, markers of adipogenic differentiation were markedly reduced by Fgf8b exposure. Conversely, preadipocyte factor 1 (Pref-1) displayed an opposite behavior in response to Fgf8b than to rosiglitazone. On the protein level, markers of terminal adipocyte differentiation hormone-sensitive lipase and perilipin were both virtually absent after Fgf8b treatment, while mitochondrial abundance marker subunit 4 of cyctochrome C oxidase displayed reduced abundance (Fig. 3C,D). Taken together, while rosiglitazone promoted both adipogenesis and brite adipocyte recruitment, Fgf8b induced Ucp1 as well as specific brite markers but interfered with adipogenesis.

**Figure 4:**
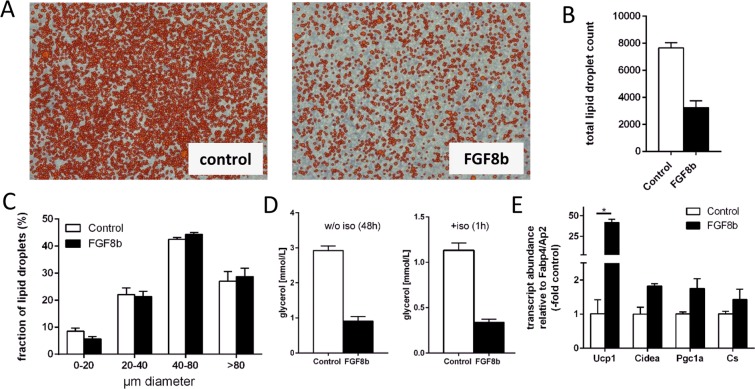
Fgf8b interferes in adipocyte differentiation. (**A**) The number of mature, lipid-laden adipocytes is reduced at the end of differentiation in a medium containing Fgf8b (125 ng/ml) versus control treated cells (con). Orange colour indicates automatic detection of a lipid droplet by an image analysis algorithm. (**B**,**C**) Final lipid droplet number is decreased (**B**), while size distribution is similar (**C**) after Fgf8b treatment. (**D**) Unstimulated (left panel) and isoproterenol-stimulated (iso, right panel) lipolytic rate determined by glycerol release are both decrease after differentiation in the presence of Fgf8b. Bars depict mean values ± SD, n = 3–4. (**E**) Transcript abundance of brown/brite adipocyte markers UCP1, Cidea and Pgc1a was increased by Fgf8b when normalized to the marker of terminal adipocytes differentiation AP2/Fabp4. Ucp1, uncoupling protein 1; Cidea, cell death-inducing DNA fragmentation factor alpha-like effector A; Pgc1a, peroxisome proliferator-activated receptor gamma, coactivator 1 alpha; Cs, citrate synthase; bars are mean values ± SD, n = 3. Stars indicate a significant difference (p < 0.05, Sidak’s multiple comparisons test).

Morphology of cells and their lipid droplets corroborated the above interpretation. The number of mature, lipid-laden adipocytes was markedly lower after differentiation in a medium containing Fgf8b (Fig. 4A,B and Supplemental Fig. 2), while their size distribution remained similar (Fig. 4C). In principal, fully differentiated adipocytes may have lost their lipid deposits due to Ucp1-mediated uncoupled respiration. However, both unstimulated and stimulates lipolysis rate was much lower in Fgf8b treated cells (Fig. 4D) and we did not detect Ucp1-mediated respiration (Fig. 2F). One possible scenario, in which Pref-1 and Ucp1 are both plausibly upregulated, is a decreased overall adipogenesis, but with induction of brite characteristics in a small subset of differentiated adipocytes. Indeed, in relation to the abundance of the adipogenic differentiation marker Fabp4/Ap2, the expression of UCP1 was increased by Fgf8b. A similar trend was observed for the brite markers Cidea, Pgc1a and Cs (Fig. 4E).

### FGF8b reprograms proliferating preadipocytes

**Figure 5:**
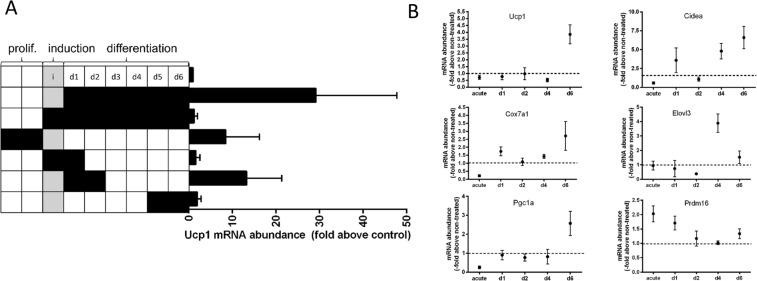
Fgf8b reprograms proliferating preadipocytes. (**A**) White adipocytes were treated with Ffgf8b (125 ng/ml) for 48 hours either during proliferation, induction, early or late differentiation and compared to treatment during the entire differentiation. Black squares indicate days of Fgf8b treatment. Treatment during the entire differentiation period led to strongest Ucp1 induction. During proliferation and early differentiation cells were most susceptible for Fgf8b treatment, while inclusion of the induction phase into the treatment abrogated its effect. Bars are mean values ± SD, n = 5. (**B**) Time course of indicated mRNA expression following a 48 hours Fgf8b treatment pulse on proliferating preadipocytes. Points are mean values ± SD, n = 3.

Treatment of preadipocytes with Fgf8b interfered with adipogenesis. This led us to study its effect on proliferating preadipocytes before induction. Fgf8b did not increase the proliferation rate (Suppl. Fig. 1A), but interfered with contact inhibition and thereby promoted denser cell growth (Suppl. Fig. 1B,D). In the previous experiments, FGF8b treatment was restricted to the differentiation phase. Therefore we probed whether the observed alteration in contact inhibition of preadipocytes is linked to the appearance of brite adipocytes later. Bone morphogenetic protein 7, for instance, promotes a re-routing of adipocyte differentiation to the brite lineage when applied for a short time window during proliferation^31^. We investigated the time course of sensitivity to Fgf8b by treating (pre-)adipocytes for 48 h during proliferation, induction and different time spans during differentiation (Fig. 5A). Recruitment of Ucp1 expression was clearly abrogated whenever Fgf8b was applied during the induction phase, while both proliferating and early differentiating preadipocytes were more responsive than late differentiated adipocytes. This pattern of Fgf8b sensitivity was not mirrored by parallel expression changes in any one of the four FGF receptor genes (Suppl. Fig. 3). While FGFR1, 2 and 3 transcripts were constantly present at a similar level during proliferation, induction and differentiation, FGFR4 displayed increased expression during early adipogenesis, but was very low expressed during proliferation.

Following a two day Fgf8b pulse on proliferating pre-adipocytes, Ucp1 transcript was increased after six days of differentiation (Fig. 5B) and accompanied by further transcripts indicative of brite adipocytes, i.e. Cidea, Cox7a1, Elovl3 and Pgc1a. Interestingly, the transcription factor PRDM16, known to play a dominant role in brite and brown cell commitment^32,33^, was acutely induced in proliferating, treated cells. We conclude that Fgf8b mainly acts on preadipocytes, predisposing them to undergo differentiation into brite adipocytes, but interfering with adipogenesis itself while present.

### FGF8b does not recruit brite adipocytes *in vivo*

**Figure 6:**
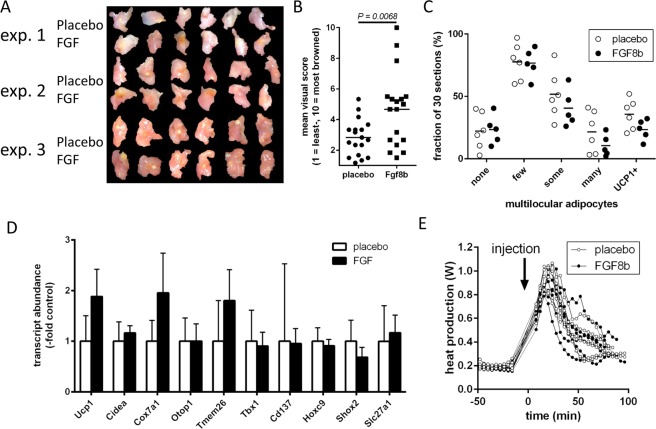
*In vivo* application of Fgf8b into epididymal white adipose. Implantation of pellets releasing 100 ng FGF8b per day and placebo pellets into the epididymal white adipose tissue results in a brownish aureole around the implantation site of FGF8b, but not of placebo pellets after three weeks of treatment. (**A**) Photographs of epididymal depots from three independent experiments of 6 mice each. (**B**) Visual browning score on a scale of 1 (least) to 10 (most) browning around a pellet was assessed by six blinded observers. Each dot represents the mean value for one specific depot, n = 18, p = 0.0068 (t test). (**C**) Visual browning assessment by keywords describing multilocular adipocyte occurrence and UCP1 immunostaining on histological sections was assessed by six blinded observers. Data is provided as percentage of 30 sections per depots that fit the keyword description. Each dot represents the mean value for one specific depot; no significant differences (Holm-Sidak multiple test). (**E**) Indirect calorimetry and injection of norepinephrine did not reveal a difference in maximal thermogenic capacity in Fgf8b versus placebo treated mice. (**D**) Transcript abundance of brite adipocyte markers genes was not markedly increased. Ucp1, uncoupling protein 1; Cidea, cell death-inducing DNA fragmentation factor alpha-like effector A; cox7a1, cytochrome c oxidase subunit 7A1; otop1, otopetrin 1; Tmem26, Transmembrane protein 26; Tbx1, T-box transcription factor 1; Cd137, cluster of differentiation 137; Hoxc9, homeobox C9; Shox2, Short stature homeobox 2; Slc27a1, Long-chain fatty acid transport protein 1; n = 5; no significant differences (Holm-Sidak multiple test).

We explored the potential of FGF8b to recruit brite adipocytes in epididymal white adipose tissue *in vivo*. The treatment with the paracrine peptide was realized by means of implantable pellets releasing 100 ng FGF8b into the surrounding interstitium. One such pellet or a placebo pellet was implanted unilaterally into the epididymal white adipose tissue of male mice. After three weeks of treatment, mice of both groups were comparable in body mass, fat depot mass and plasma chemistry indicating no systemic, adverse effects of local treatment in one fat depot by a paracrine agent (Suppl. Fig. 4). Visual inspection of the implantation site revealed an area of distinct brown color surrounding the implanted FGF8b release pellets, but not the placebo pellets (Fig. 6A,B). Surprisingly, we did not detect any change in the number of multilocular adipocytes or UCP1 immunoreactivity in histological sections of the implanted depot (Fig. 6C). Furthermore, transcript abundance of brite adipocyte markers genes were only slightly or not at all increased (Fig. 6D). Primary mature adipocytes from treated tissue did not display an increase in respiratory complex IV capacity as indicative for an increased brite cell abundance^34^, nor did we detect increased respiration with the preferred respiratory substrate of brown adipocytes glycerol-3-phosphate or increased mitochondrial mass as manifested in increased citrate synthase activity (Table 1). Consequently, injection of norepinephrine did not lead to an altered, thermogenic response (Fig. 6E).

**Table 1:** Biochemical assessment of mitochondrial function in epididymal adipose tissue explants from the vicinity of implanted FGF8b release or placebo pellets.

		Placebo	Fgf8b	p
1	adipocyte respiration on G3P (nmol O_2_/s*g tissue)	0.84 ± 0.30	0.86 ± 0.36	0.923
2	cytochrome C oxidase activity (nmol O_2_/s*g tissue)	3.93 ± 1.64	5.24 ± 2.36	0.289
3	citrate synthase activity (arbitrary unit/g tissue)	1.12 ± 0.51	1.36 ± 0.41	0.396
4	ratio of 1 to 2	0.22 ± 0.04	0.17 ± 0.02	0.016
5	ratio of 1 to 3	0.82 ± 0.31	0.63 ± 0.20	0.244
6	ratio of 2 to 3	3.74 ± 1.42	3.81 ± 1.23	0.935

1 - Respirometry of isolated, mature adipocytes with glycerol-3-phosphate (G3P) as substrate. 2–Cytochrome C oxidase activity in tissue homogenates. 3 – Citrate synthase activity in tissue homogenates. 4–6 – Ratios between 1–3. None of the parameters assessed was significantly different (Sidak’s multiple comparisons test), n = 6.

In summary, while FGF8b caused an unexplained, brown aureole around the site of its release, no other evidence for the increased number of brite adipocytes or increased respiratory capacity could be verified. By the chosen application route and release rate, FGF8b did not recruit brite adipocytes *in vivo*, while it led to expression of UCP1 in cultured white (pre-)adipocytes.

## Discussion

Fibroblast growth factors (FGFs) are a heterogeneous group of proteins encompassing endocrine hormones, paracrine peptides as well as intracellular signaling components^12^. Endocrine FGF21 is a key regulator of substrate utilization and is able to recruit brite adipocytes in white adipose tissue^10,11^. Both endocrine and paracrine FGFs bind to the same set of FGF receptors either stabilized by a klotho cofactor (endocrine FGFs) or by heparin. We explored the possibility that a paracrine FGF may recruit brite adipocytes by a similar route, but with the benefit of local applicability and in the absence of systemic effects. In an initial screening we identified FGF8b to increase uncoupling protein 1 (UCP1) transcript and protein abundance in cultured white adipocytes.

From the data presented in this study it is obvious that the mode of action of FGF8b is neither rerouting differentiation of white towards brite adipocytes nor transdifferentiation of white adipocytes. FGF8b inhibits adipogenesis and does not lead to the appearance of multilocular brite adipocytes *in vivo*. The visual browning of adipose tissue and a trend towards increased brite adipocyte marker transcript abundance appeared suggestive, but were not accompanied by an increase in multilocular cells or mitochondrial enzyme activity. Possibly, our choice of dose and delivery method led to a marginal effect size. Another plausible, not yet considered explanation for the increased abundance of UCP1 transcript and protein despite reduced adipogenesis is that the cells expressing UCP1 are in fact not mature adipocytes, but preadipocytes. Although an unconventional interpretation, others have reported a similar phenomenon in response to a paracrine FGF^35^. The observed UCP1 expression may thus originate from preadipocytes or at least not fully differentiated adipocytes that atypically express this marker of terminal differentiation along with other brite identity markers (e.g. Cd137), but without being accompanied by classical brown or adipogenesis markers.

As Kahn and Tseng point out in their recently published patent reporting a similar effect of paracrine FGF6, it reveals preadipocytes as a possible target structure to recruit energy-consuming cells by pharmacological intervention^35^. The lack of Ucp1-mediated respiration in our assays and the lack of brite cell recruitment in our vivo study exemplify the challenges entailed in exploiting this concept in practice. In any case, this unusual phenomenon represents a unique chance to dissect transcriptional regulatory networks targeting specifically UCP1 transcription from higher order ones governing classical brown adipogenesis. Intriguingly, several markers of brite adipocytes (Tmem26, Tbx1, Cd137) appear to be part of the same gene set. The role of these genes in adipocyte identity is not entirely clear. On the one hand, they associate with brite adipocyte abundance on the depot level, but on the other hand, they are not increased by cold or rosiglitazone, two stimuli clearly increasing brite adipocyte number per depot^27,36,37^. These seemingly conflicting findings plausibly fit together with a role as markers of brite preadipocytes, not mature thermogenic brite adipocytes.

At present, we do not know the intracellular pathway connecting FGF8b and the UCP1 promoter. Any one of the seven splice variants of four FGF receptor genes may mediate this effect^38^. By its expression pattern during differentiation, Fgfr4 seemed less important (Suppl. Fig. 3) while the strong changes in transcript abundance in response to Fgf8b treatment of Fgfr1 (upregulated) and Fgfr2 (downregulated) (Fig. 3A) support a role for one their gene products. Downstream of these receptors, several parallel and/or alternative intracellular signal transduction pathways emerge, any one or several of which may converge on the UCP1 gene^39^. We will dissect these regulatory networks in future work.

Taken together, we report FGF8b to be a paracrine signal leading to specific upregulation of UCP1 in murine preadipocytes of epididymal origin. The unique presence of UCP1 in a cell other than a mature brown or brite adipocyte represents a promising model to dissect UCP1-specific transcriptional regulation.

## Supplementary information

Supplementary Information

## Data Availability

The datasets generated and analyzed during this study are available from the corresponding authors on reasonable request.
