# Supplementary material for: Fibroblast growth factor 8b induces uncoupling protein 1 expression in epididymal white preadipocytes
Source: Sci Rep. 2019 Jun 11;9:8470. doi: 10.1038/s41598-019-44878-w (PMC6560125; doi:10.1038/s41598-019-44878-w)
Supplement: Supplementary file 1 — Supplementary Information [file 41598_2019_44878_MOESM1_ESM.pdf]

Supplemental Information

Fibroblast growth factor 8b induces uncoupling protein 1 expression in epididymal white preadipocytes

Sören Westphal[1*], Thomas Gantert[2], Caroline Kless[2], Kristina Hüttinger[2], Martin Klingenspor[2,3,4] and Tobias Fromme[2*]

[1]: Department of Internal Medicine II, University of Ulm, Ulm, Germany
[2]: Chair of Molecular Nutritional Medicine, TUM School of Life Sciences, Technical University of Munich, Freising, Germany
[3]: EKFZ - Else Kröner-Fresenius Center for Nutritional Medicine, Technical University of Munich, Freising, Germany
[4]: ZIEL - Institute for Food & Health, Technical University of Munich, Freising, Germany

*corresponding authors:

Dr. Tobias Fromme
Technical University of Munich
Chair of Molecular Nutritional Medicine
Gregor-Mendel-Str. 2
85354 Freising, Germany
Tel. +49.8161.71.3850
fromme@tum.de

Dr. Sören Westphal
Department of Internal Medicine II
University of Ulm
Albert Einstein Allee 33
89081 Ulm, Germany
Tel. +49.171.3213685
soeren.westphal@t-online.de

Supplemental Figure 1

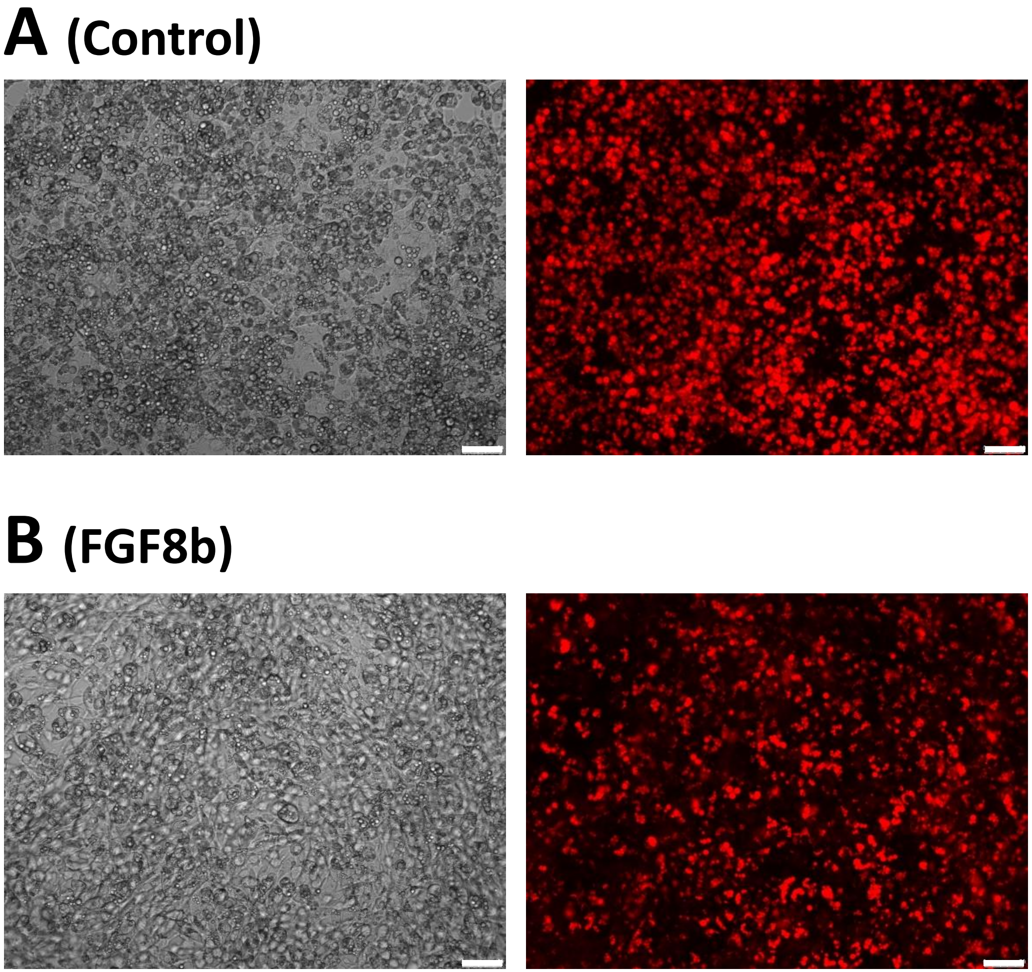

Supplemental Figure 1 – Cellular morphology is altered by Fgf8b treatment. Representative image of differentiated adipocytes treated with 125ng/ml Fgf8b during six days of differentiation (A) or not (B). Left panels are bright field images, right panel show a fluorescent BOPIDY stain to visualize intracellular lipid. All scale bars = 50µm.

Supplemental Figure 2

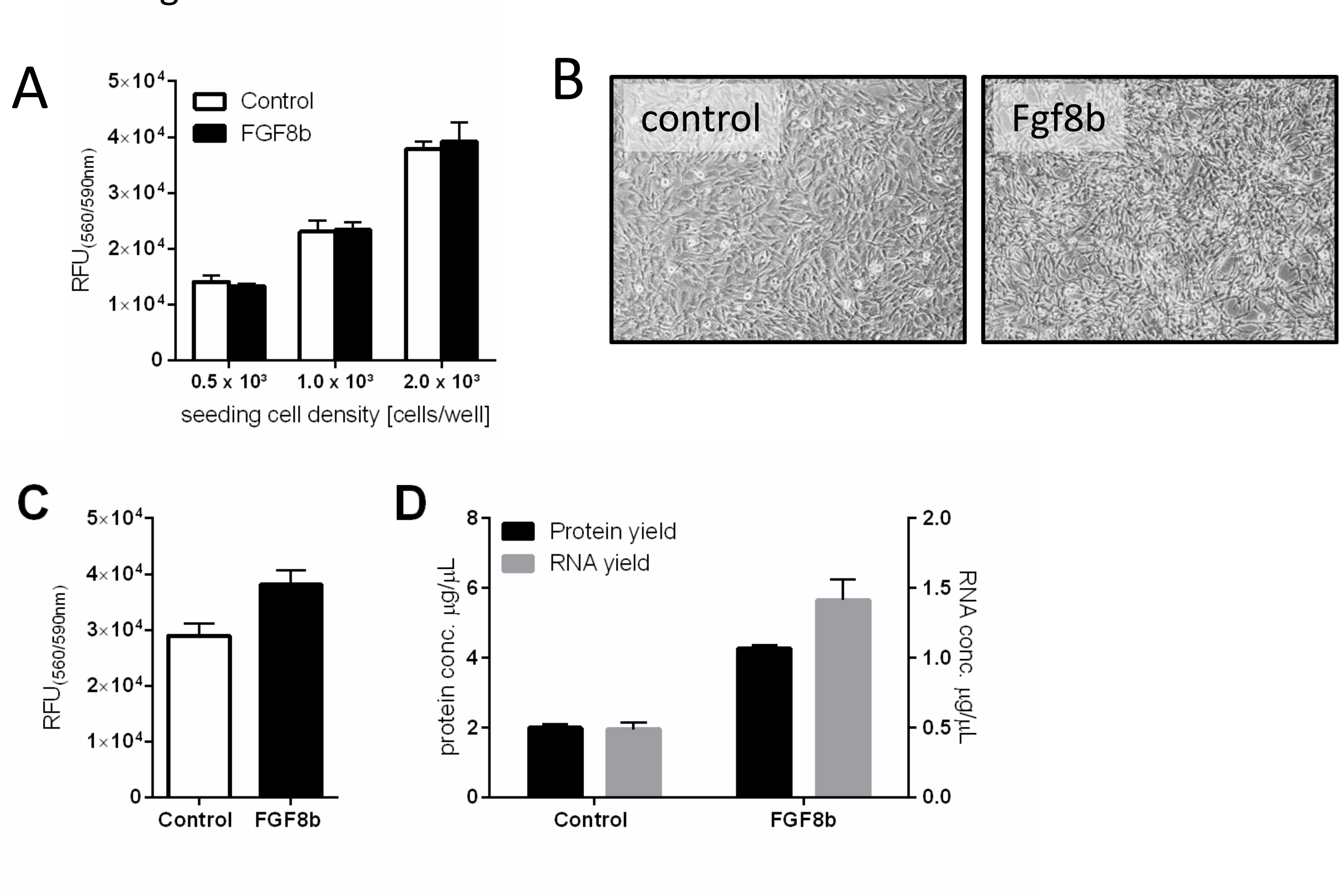

Supplemental Figure 2 - Fgf8b did not increase proliferation rate, but abrogated contact inhibition. A - Resazurin reduction after 4h of exposure as a proxy of cell number 48h after seeding preadipocytes in different initial densities. Proliferation rate is not altered in the subconfluent state. B – Representative images of proliferating preadipocytes 2 days after first reaching confluency in the absence or presence of 125ng/ml FGF8b. C - Resazurin reduction after 4h of exposure as a proxy of cell number 48h after first reaching confluency. Cells grow much denser in the presence of Fgf8b. D – Both protein and RNA amount isolated from preadipocytes 48h after first reaching confluency is increased by Fgf8b exposure.

Supplemental Figure 3

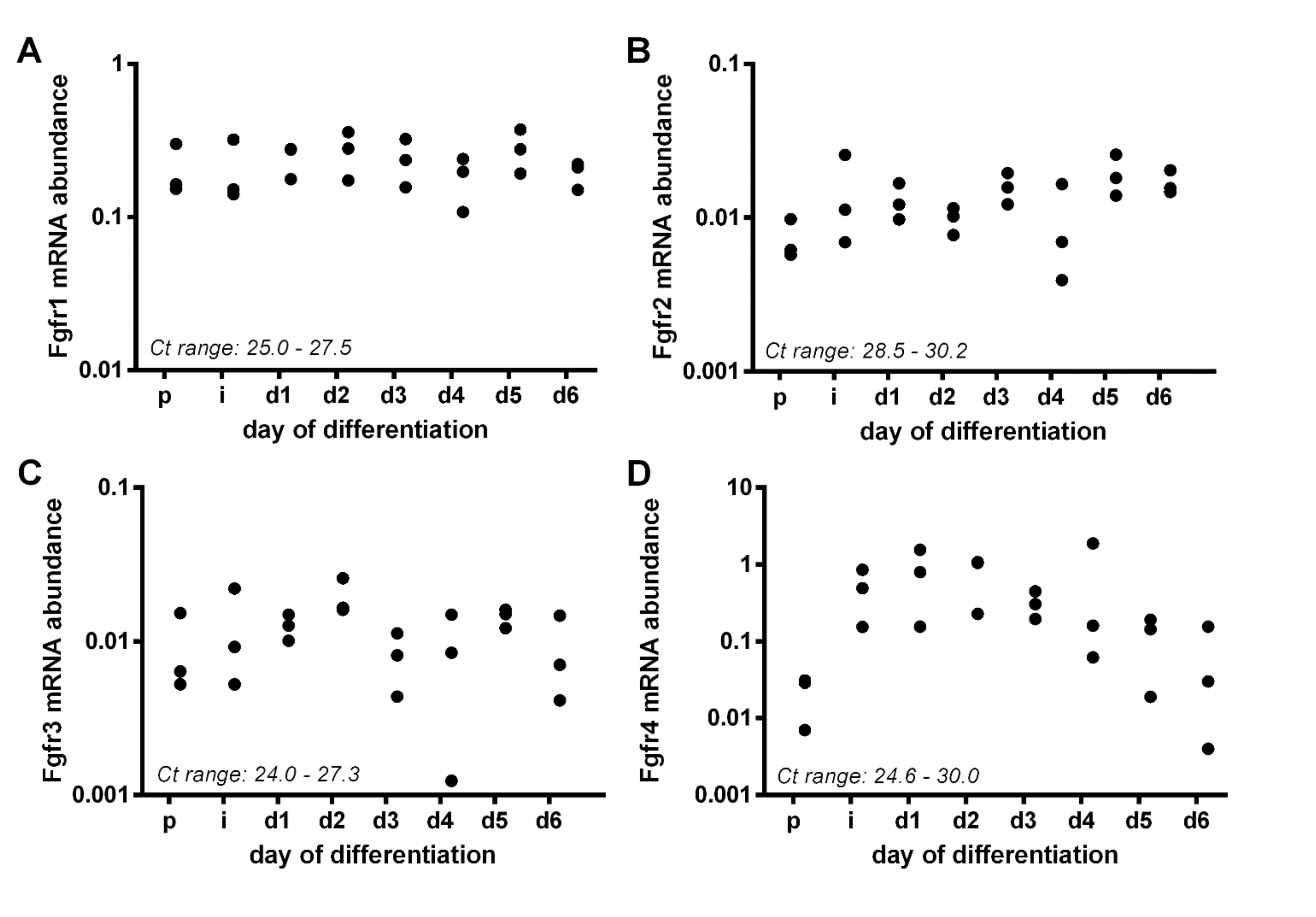

Supplemental Figure 3 Transcript abundance of fibroblast growth factor receptors during adipocyte differentiation. We determined transcript abundance of fibroblast growth factors (Fgfr) 1 to 4 in cultured white adipocytes including proliferating (p) cells, cells during induction (i) and differentiation (d followed by number of differentiation day). All transcripts were normalized to heat shock protein 90 (Hsp90) abundance, n=3-6. We provided cycle time (Ct) ranges as a surrogate for absolute expression differences between receptors.

Supplemental Figure 4

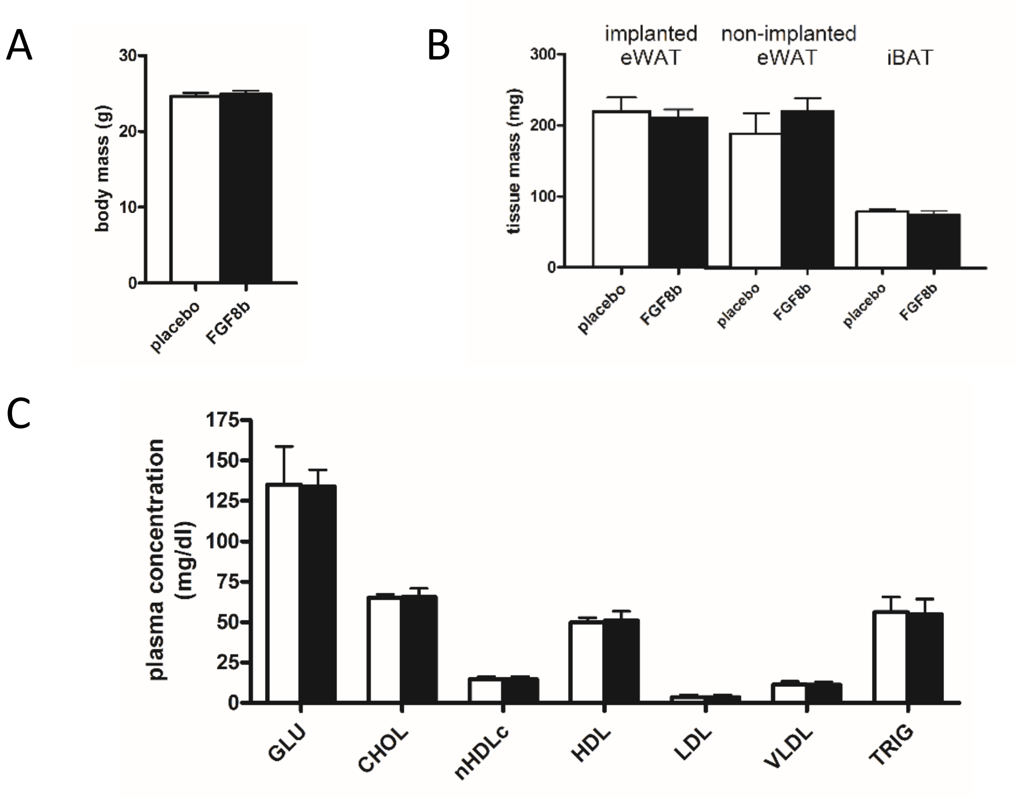

Supplemental Figure 4 - Physiological data of mice implanted with Fgf8b release or placebo pellets. A - Body mass 3 weeks post-implantation. B - Masses of implanted epididymal, non-implanted contralateral epididymal and interscapular brown fat depot. C - Plasma metabolites of carbohydrate and lipid metabolism were not affected by Fgf8b treatment. GLU - glucose, CHOL - total cholesterol, nHDLc - non-HDL cholesterol, HDL - high density lipoproteins, LDL - low density lipoproteins, VLDL - very low density lipoproteins, TRIG – triglycerides.

Supplemental Figure 5

Western Blot shown cropped in Figure 1D

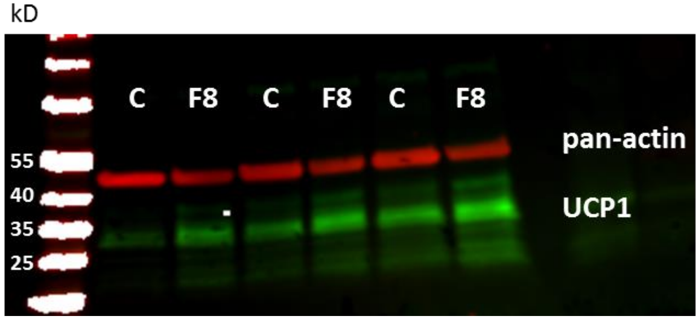

C = control, F8 = Fgf8b treated. Shown is the entire area scanned (Odyssey Imaging System, Licor).

Western Blot shown cropped in Figure 3C

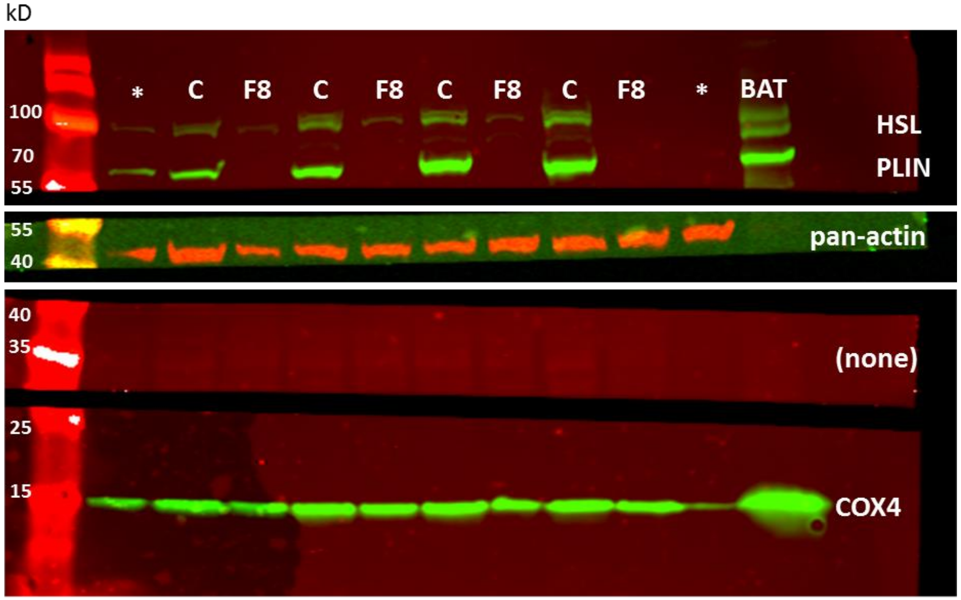

The blot was physically cut into four section and each incubated with different combinations of primary and secondary antibody. C = control, F8 = Fgf8b treated, BAT = brown adipose tissue protein lysat, * = unrelated samples. Shown is the entire area scanned (Odyssey Imaging System, Licor).

Supplemental Table 1

target	primer 1	primer 2	reference
Actb	AGAGGGAAATCGTGCGTGAC	CAATAGTGATGACCTGGCCGT	
Cd137	CGTGCAGAACTCCTGTGATAAC	GTCCACCTATGCTGGAGAAGG	Wu, J. et al.; Cell 150, 366-376 (2012)
Cidea	TGCTCTTCTGTATCGCCCAGT	GCCGTGTTAAGGAATCTGCTG	
Cox7a1	CCGACAATGACCTCCCAGTA	TGTTTGTCCAAGTCCTCCAA	
Cs	CTGAGGAAGACTGACCCTCG	TTCATCTCCGTCATGCCATA	
Elovl3	TCCGCGTTCTCATGTAGGTCT	GGACCTGATGCAACCCTATGA	
Fabp4	GATGGTGACAAGCTGGTGGT	TTTATTTAATCAACATAACCATATCCA	
Fasn	GCATTCAGAATCGTGGCATA	TTGCTGGCACTACAGAATGC	
Fgf21	GTGTCAAAGCCTCTAGGTTTCTT	GGTACACATTGTAACCGTCCTC	
Fgfr1	CCACCAACTGCTTGAACGTA	CCGGATCTACACACACCAGA	
Fgfr2	AAACACAGAATCGTCCCCTG	AGGGACACAGGATGGACAAG	
Fgfr3	TGAGGATGCGGTCTAAATCC	ACCGAGTCTACACCCACCAG	
Fgfr4	AAGGAGAGTGGTCCTTCGCT	GTGGCTGTGAAGATGCTGAA	
Gtf2b	GCTGTGGAACTGGACTTGGT	AGTTTGTCCACTGGGGTGTC	
Hoxc9	GCAGCAAGCACAAAGAGGAGAAG	GCGTCTGGTACTTGGTGTAGGG	Walden, T. B. et al., Am J Physiol Endocrinol Metab 302, E19-31, (2012).
Hsl	GCTTGGTTCAACTGGAGAGC	GCCTAGTGCCTTCTGGTCTG	
Hsp90	AGGAGGGTCAAGGAAGTGGT	TTTTTCTTGTCTTTGCCGCT	
Klb	ATGTCCAGGAGGCTCTGAAA	AGCAAATGGTGCAGTCTGTG	Wu, J. et al.; Cell 150, 366-376 (2012)
Otop1	GGACCTGATGCAACCCTATGA	ACCATGCTCTACGTGCTGTG.	
Pparg	TCAGCTCTGTGGACCTCTCC	ACCCTTGCATCCTTCACAAG	
Ppargc1a	GGACGGAAGCAATTTTTCAA	GAGTCTTGGGAAAGGACACG	
Prdm16	CTGTTAGCTTTGGAGCCGAC	GACGAGGGTCCTGTGATGTT	
Pref1	GGAGGCTGGTGATGAGGAGATC	AGAGCTCTAAGGAACCCCGGTA	
Shox2	TGGAACAACTCAACGAGCTGGAGA	TTCAAACTGGCTAGCGGCTCCTAT	Walden, T. B. et al., Am J Physiol Endocrinol Metab 302, E19-31, (2012).
Slc27a1	CTGGGACTTCCGTGGACCT	TCTTGCAGACGATACGCAGAA	Wu, J. et al.; Cell 150, 366-376 (2012)
Tbx1	GGCAGGCAGACGAATGTTC	TTGTCATCTACGGGCACAAAG	Wu, J. et al.; Cell 150, 366-376 (2012)
Tmem26	ACCCTGTCATCCCACAGAG	TGTTTGGTGGAGTCCTAAGGTC	Wu, J. et al.; Cell 150, 366-376 (2012)
Ucp1	TCTCTGCCAGGACAGTACCC	AGAAGCCCAATGATGTTCAG	
